# Ice-Template Crosslinked PVA Aerogels Modified with Tannic Acid and Sodium Alginate

**DOI:** 10.3390/gels8070419

**Published:** 2022-07-05

**Authors:** Lucía G. De la Cruz, Tobias Abt, Noel León, Liang Wang, Miguel Sánchez-Soto

**Affiliations:** 1 Centre Català del Plàstic, Universitat Politècnica de Catalunya Barcelona Tech (EEBE-UPC), Av. d’Eduard Maristany, 16, 08019 Barcelona, Spain; lucia.de.la.cruz@upc.edu (L.G.D.l.C.); tobias.abt@upc.edu (T.A.); noel.leon@upc.edu (N.L.); 2 Key Laboratory of Advanced Textiles Composites of Ministry of Education, Tiangong University, Binshui West Road 399, Xiqing District, Tianjin 300387, China; liangwang@tiangong.edu.cn

**Keywords:** aerogel, tannic acid, fire resistance, crosslinking, lyophilization

## Abstract

With the commitment to reducing environmental impact, bio-based and biodegradable aerogels may be one approach when looking for greener solutions with similar attributes to current foam-like materials. This study aimed to enhance the mechanical, thermal, and flame-retardant behavior of poly(vinyl alcohol) (PVA) aerogels by adding sodium alginate (SA) and tannic acid (TA). Aerogels were obtained by freeze-drying and post-ion crosslinking through calcium chloride (CaCl_2_) and boric acid (H_3_BO_3_) solutions. The incorporation of TA and SA enhanced the PVA aerogel’s mechanical properties, as shown by their high compressive specific moduli, reaching up to a six-fold increase after crosslinking and drying. The PVA/TA/SA aerogels presented a thermal conductivity of 0.043 to 0.046 W/m·K, while crosslinked ones showed higher values (0.049 to 0.060 W/m·K). Under TGA pyrolytic conditions, char layer formation reduced the thermal degradation rate of samples. After crosslinking, a seven-fold decrease in the thermal degradation rate was observed, confirming the high thermal stability of the formed foams. Regarding flammability, aerogels were tested through cone calorimetry. PVA/TA/SA aerogels showed a significant drop in the main parameters, such as the heat release rate (HRR) and the fire growth (FIGRA). The ion crosslinking resulted in a further reduction, confirming the improvement in the fire resistance of the modified compositions.

## 1. Introduction

Natural porous materials such as cork, wood, bones, or sponges are lightweight materials that are well known and historically employed. However, it was not until the 1960s that science and technology were engaged in producing synthetic foams. The terms “foam” and “porous” are often used interchangeably. However, they involve different concepts as foam implies volume expansion, while porous material does not. The need to find lightweight, sustainable materials to replace conventional foams has encouraged academia and industry’s interest in using biodegradable and bio-based polymer sources.

Aerogels are ultra-light, highly porous materials characterized by a large specific surface area, which makes them excellent candidates for absorbing media [1], energy storage [2], or thermal insulation applications [3]. Among the different types, inorganic silica aerogels are probably the most studied. However, silica aerogels are limited by their brittleness and low compressive properties; therefore, organic and organic-inorganic hybrid aerogels have emerged as a mechanically tough alternative to inorganic aerogels [4].

Poly(vinyl alcohol) is a synthetic polymer with non-toxic, chemical resistant, and biodegradable properties. It can be easily dissolved in water, making it a suitable candidate to make hydrogels with water as the only solvent. However, the limited mechanical properties, low thermal stability, and poor fire resistance have restricted the potential application of PVA-based aerogels. Common approaches to increase these properties have been, among others, the use of inorganic fillers [5], crosslinking [6], or the generation of oriented structures through directional freezing [7]. Natural substances can be employed to generate fully biodegradable and mechanically strong aerogels with improved properties. Alginates are hydrophilic polysaccharides extracted from seaweed that can yield mechanically robust structures, as reported by Li et al. [8]. Alginates also showed high resistance to flame and did not produce harmful gases during burning [9,10]. On the other hand, tannic acid is a natural polyphenolic compound that contains five digallic acid units connected through ester linkages to a central glucose core. Tannic acid is known to form ionic, hydrophobic, or hydrogen interactions with other polymers such as PVA and is considered a non-toxic flame retardant charring agent [11]. Therefore, these two components can be combined to improve the mechanical and fire resistance of PVA and create bio-aerogels that can be used as potential substitutes for traditional polymer-based foams such as polyurethanes or expanded polystyrene.

Tannic acid has been previously employed in combination with PVA to create high-performance materials. For instance, Chen et al. [12] prepared shape memory PVA/TA hydrogels with enhanced mechanical properties combining the strong, permanent hydrogen bonding between TA and PVA and the weak, temporary hydrogen bonding between chains. Guan et al. [13] also used TA as a crosslinker for PVA films that exhibited excellent tensile strength and fracture toughness with an optimal TA concentration of 3% wt. Regarding aerogels, Cheng et al. [14] prepared PVA-TA aerogels in which TA increased the compressive modulus and decreased the combustion parameters compared to pure PVA.

The present work aims to demonstrate the possibilities of using natural compounds to enhance PVA-based aerogels’ properties, making them environmentally friendly biodegradable alternatives to conventional foams. Taking advantage of the abundant hydroxyl groups in the PVA backbone, in this study, PVA has been modified with TA and sodium alginate (SA), followed by a post-ion-crosslinking with calcium chloride (CaCl_2_) and boric acid (H_3_BO_3_). Ca^2+^ and B(OH)_4_^−^ ions have been reported to interact with SA via the egg box model [15] and by forming the PVA-boric acid complexes [16]. Together, these modifications enhance the mechanical and fire resistance properties by establishing bonding between components. Several aerogel compositions have been analyzed in the following manuscript in terms of morphology, thermal, mechanical properties, and fire behavior. To our knowledge, this combination of materials has not previously been studied.

## 2. Results and Discussion

The denomination of samples under study is according to the constituents used, followed by their respective content in 100 mL of aqueous solution (See Table S1). For instance, P5T2A3 means that 5 g of PVA, 2 g of TA, and 3 g of SA have been dissolved in 100 mL of deionized (DI) water. NaOH was also added until a pH of 8.5 was reached in the corresponding precursor solution. The addition of NaOH was necessary to overcome the high hydrogen bonding attraction between TA and PVA that causes a fast precipitation of a solid. NaOH can deprotonate the phenolic groups of TA (pK_a_ = 8.5) [17], increasing the solubility of the components [14].

### 2.1. Density and Porosity

The results of the apparent densities ($\rho_{app}$), theoretical ($P_{th}$), and experimental porosities ($P_{exp}$) of the aerogels produced are listed in Table 1. As can be observed, after incorporating SA and TA into the aerogel matrix, the apparent densities increased around two-fold compared to the P5 reference. This increment was, to some extent, expected since SA (1.695 g/cm^3^) and TA (1.560 g/cm^3^) powder densities are higher than that of PVA (1.281 g/cm^3^). However, the main reason for the change in density was associated with the higher number of solids in the precursor solutions of the modified aerogels. Regarding porosities, the higher the percentage of TA and SA in the aerogel matrix, the lower the obtained void fraction. The increase in the number of solids in the precursor gel was, as well, the reason for the decrease in porosity of the resultant PVA/TA/SA blends. The driving force to create the aerogel structure comes from ice generation during freezing. When the temperature decreases and ice is created from the solution, it reaches an oversaturated state. Under this condition, the excess solute is expelled outwards at the frontiers of the growing ice. A higher number of solids dissolved in the precursor solution implies greater difficulty for the ice to grow and expand, hence lower porosity in the final aerogel after ice sublimation. The resultant porous structure is considered to be open type because it corresponds to the space occupied by the sublimated ice.

As expected, the densities of the PVA/TA/SA aerogels increased after crosslinking. The presence of B(OH)_4_^−^ ions led to complexation with the PVA diol groups and TA, leading to a more closed-cell structure. In addition, Ca^2+^ ions interacted with the alginate chains causing the well-known egg-box conformation. As the main component of aerogels was PVA, the highest shrinkage was found in the samples with the higher relative amount of this polymer (P5T2A1 and P5T2A2) after its reaction with borate ions.

### 2.2. Morphology

**Figure 1:**
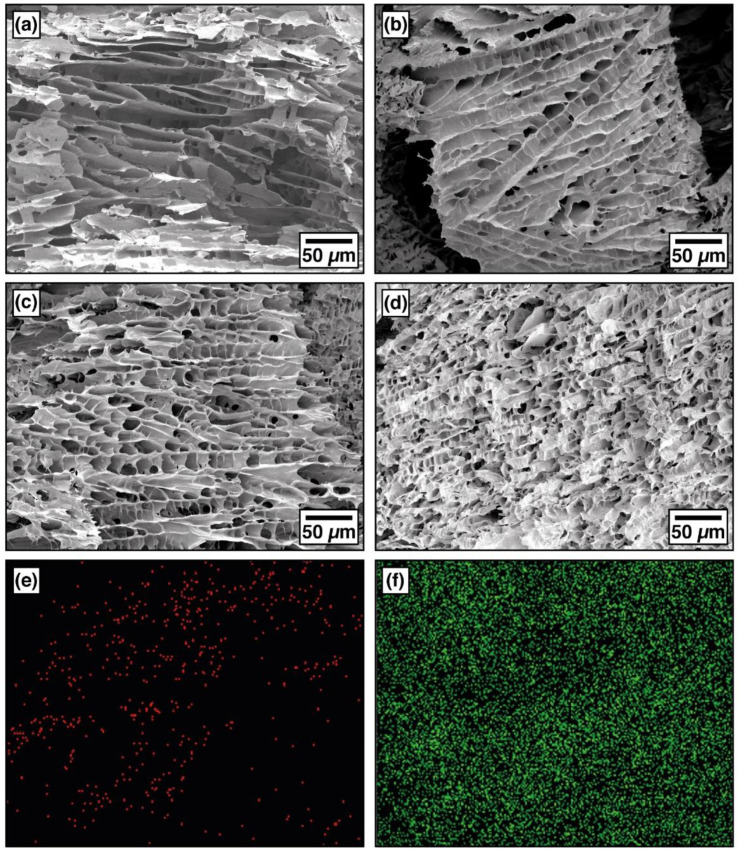
SEM micrographs of uncrosslinked aerogels (**a**) P5T2A1, (**b**) P5T3A2, (**c**) P5T3A3, and (**d**) P5T2A1 crosslinked aerogel; EDS images for (**e**) boron and (**f**) calcium dispersion in P5T2A1 crosslinked aerogel.

The freeze-drying of the different molded gels yielded stable cylindrical-shaped monoliths and rectangular samples such as the ones represented in Figure S1. Their morphology was characterized through SEM observation of representative samples obtained by cryogenic cuts performed in a vertical direction. As the corresponding molds were dipped into a solid CO_2_/ethanol solution at −80 °C, the main freezing direction and ice growth went mainly from the mold boundary towards the center, in the radial direction, which corresponds to the direction of the holes in the images shown in Figure 1.

The morphology of the aerogels is dictated by the freezing of water and ice growth. At the relatively low freezing temperatures used, the crystal nucleation rate exceeded the crystal growth rate, and a large number of ice crystals were formed. The fast heat transfer led to the formation of a layered structure of columnar ice crystals [18]. As in all cases, the freezing conditions were kept constant. The main factor controlling the differences in morphology between samples was the ice growth, which is a direct function of the viscosity of the precursor gels. In particular, the viscosity of the solutions was dependent on the alginate concentration as it was the most viscous substance when dissolved in water. As can be seen, the morphological structures of the aerogels are characterized by a porous layered structure linked by struts. A certain amount of inhomogeneity can be observed in both the direction and distribution of the layers and a decrease in pore size with increasing SA content. Under a bidirectional freezing process, a highly viscous solution affects the rearrangement of the polymeric chains between the growing ice crystals. The high viscosity decreases the mobility of water molecules and retards the ice growth because it delays the rate at which mass is diffused away across the ice crystal/liquid interface leading to a decrease in pore size and the generation of a more irregular structure [19]. The amount of TA seemed not to have influenced the morphology (see Figure S2).

### 2.3. FTIR-ATR Spectroscopy

**Figure 2:**
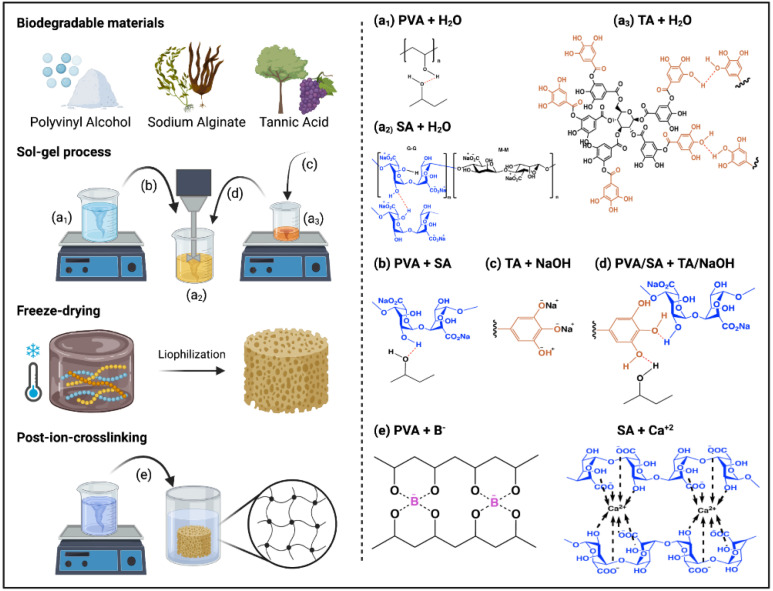
Schematic representation of the aerogels’ preparation process and the possible reactions between PVA/TA/SA aerogels and post-ion-crosslinking with CaCl_2_ and H_3_BO_3_ (Created with Biorender.com Agrmt. No. EV240EKOH).

Figure 2 shows the aerogels’ route preparation and the possible reactions between the precursors and crosslinkers.

**Figure 3:**
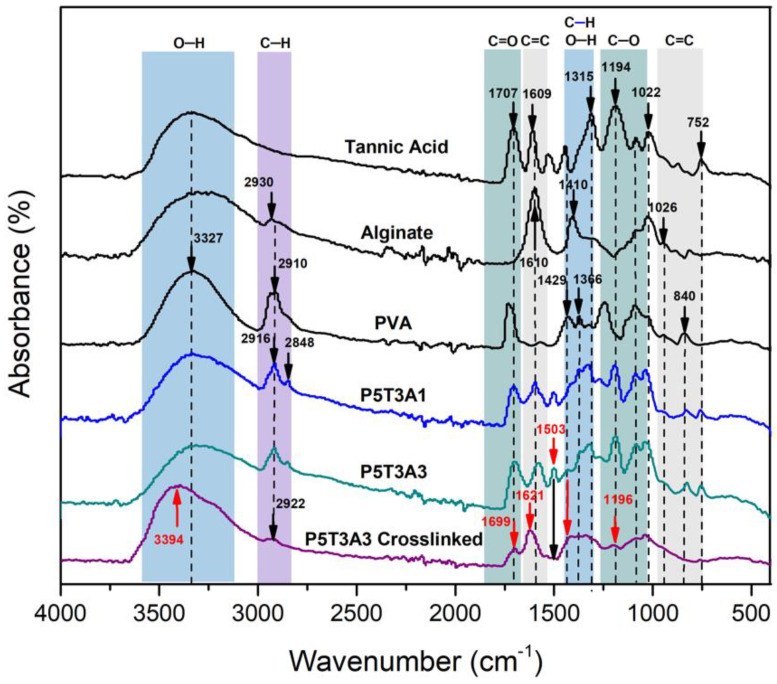
FTIR-ATR spectra of PVA, TA, SA, P5T3A3 uncrosslinked and crosslinked aerogels.

Figure 3 shows the FTIR spectra of the precursors, PVA/TA/SA uncrosslinked and crosslinked representative aerogels (the rest of the spectra are depicted in Figure S3).

The absorbance spectra of PVA, TA and SA showed a broadband between 3000 cm^−1^ to 3600 cm^−1^, corresponding to hydroxyl groups (–OH stretching mode). The –OH signal from PVA/TA/SA aerogels displayed a redshift to 3332 cm^−1^ compared to the precursors. The significant displacement confirms the hydrogen bond formation [20]. The PVA/TA/SA aerogels presented a reduction in the intensity of the –CH stretching signal characteristic of the alkanes (from PVA and SA), which appeared at 2916 cm^−1^ in P5T3A1 composition. Likewise, it is possible to observe the appearance of a –CH new peak at 2848 cm^−1^, which belongs to the increment of TA in the composition.

Furthermore, the C=O stretching from residual acetate groups from the partially hydrolyzed PVA [21], and esters from TA showed a reduction in its absorption, and the band shifted to 1707 cm^−1^. Moreover, the peaks detected at 1609, 1503, and 1040 cm^−1^ indicate the presence of C=C stretching due to TA benzene moieties [22,23]. The wavelengths at 1609 and 1410 cm^−1^ correspond to COO, and the one at 1043 cm^−1^ corresponds to C–O stretching from SA [21,24]. Furthermore, there are four C–O stretching signals from TA located at 1315, 1194, 1022, and 1085 cm^−1^ [21]. Finally, two characteristic peaks can be observed in the fingerprint region at 840 and 752 cm^−1^. The first one was attributed to the overlapping of mannuronate from SA [25] and C–C stretching vibration from PVA [26], and the second one was attributed to C–H out of plane from TA [27].

After crosslinking, the –OH band shape changed, became wider, and showed a prominent peak at 3493 cm^−1^ due to the reduction of –OH groups participating in hydrogen bonding. Moreover, a shift to small wavelengths (3200 cm^−1^) was attributed to –OH linked to higher molecular weight structures resulting from the crosslinking. The reactions of PVA/B^−^ and SA/Ca^2+^ are based on the di-diol model [16] and egg-box model [15], respectively.

The C–H– signal in P5T3A3 at 2947 cm^−1^ almost disappeared because the egg-box structure created between SA and Ca^2+^ limited the stretching vibration of the SA ring [28]. Moreover, the C–O band at ~1022 cm^−1^ became weaker due to the C–O–Ca–C–O interaction formation [28]. The signals at 1707 and 1600 cm^−1^ presented a blueshift to 1730 and 1610 cm^−1^ due to Ca^2+^ complexation [21]. The –OH and C–O moieties from TA also interacted with calcium and borate ions since their bands were displaced and decreased [29,30]. Furthermore, the decreased intensity of the peaks at 1096 cm^−1^ indicated the reduction of PVA’s crystallinity as a result of the complexation with borate ions [31]. The band at 1409 cm^−1^ was attributed to B–O stretching vibration [32]. Since the solutions were adjusted with NaOH to pH 8.5, the samples’ alkalinity allowed the chemical connections between B(OH)^−4^ and –OH from PVA and TA, generating a dense network [31]. All the signals found in the PVA/TA/SA aerogels confirmed the presence of every precursor in the sample structure as well as the crosslinking effect.

### 2.4. Mechanical Properties

**Figure 4:**
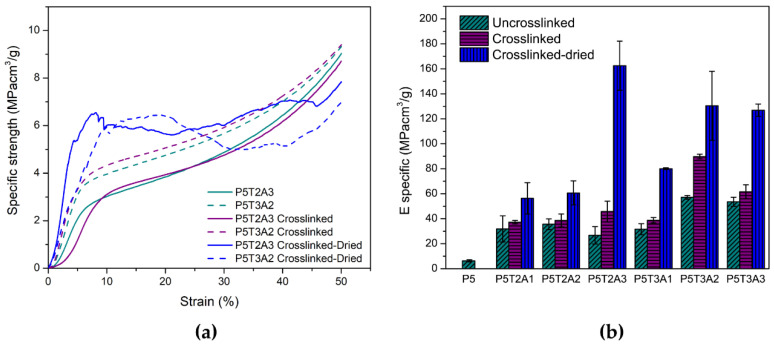
(**a**) Compressive behavior of aerogels; (**b**) comparison of the specific moduli obtained in each composition of aerogels in different states (uncrosslinked, crosslinked, and dried crosslinked).

Under compression, PVA/TA/SA aerogels exhibited similar behavior to conventional foams (see Figure 4a). Initially, the behavior was linear and elastic until it reached the yield point, followed by a constant stress plateau corresponding to plastic deformation. Later at high strains, the collapse of the pores and densification took place. The resultant characteristic values of specific Young’s modulus and stress at yield can be found in Table 2. The presence of TA increased the mechanical response under compression, creating more robust aerogels. Keeping the content of PVA and SA constant, an increase in TA content led to higher Young’s modulus and yield stress. This fact is consistent with the behavior observed by other researchers [9,14] and was attributed to hydrogen bonding attraction between components as the effect of the amount of solids is corrected by the density in the specific values. The presence of moderate amounts of SA also contributed to enhancing the mechanical characteristics. However, a decrease in the growing tendency of Young’s modulus was found when its concentration exceeded 25 wt% (samples P5T2A3 and P5T3A3). Although all components are compatible, the interaction between TA and PVA limits the interaction between PVA and SA. Therefore, above a certain concentration of SA, it is believed that excess solid content disrupts the aerogel structure, by decreasing the degree of entanglement and resulting in a loss of stiffness. Regarding pure PVA, a nine-fold increase in Young’s modulus and a ten-fold increase in the stress at yield was found for the P5T3A3 composition. Crosslinking caused a further increase in both the specific Young’s modulus and specific yield stress because of the stronger interactions and links created between the different components, especially in PVA and SA phases.

Since the base polymers and the natural modifiers used are hygroscopic materials, the samples were tested after stabilizing under 50% relative humidity. Nevertheless, it was thought relevant to determine the amount of moisture absorbed by the samples. PVA/TA/SA composites absorbed 11% of moisture, whereas crosslinked samples presented an interval of moisture uptake between 25 and 34%. This difference is attributed to the created 3D network structure, which allows effective immobilization of water molecules inside the aerogel. Additionally, since CaCl_2_ and H_3_BO_3_ are highly hygroscopic, they also increase moisture uptake. The highest amount of moisture was found in the P5T2A3 composition, which also had the highest percentage of SA.

In PVA, water absorption causes plasticization as well as glass transition temperature (Tg) and mechanical property depression [33]. Therefore, the crosslinked samples were also tested after being dried in an oven to determine the effect of absorbed water on mechanical properties. Results are depicted in Figure 4b. The dried crosslinked samples showed a much higher specific Young’s modulus than the conditioned ones, reaching the maximum in the P5T2A3 composition, which also had the highest moisture absorption. Mechanical properties in the dried state increased with the amount of alginate added to the blend (see Table 2).

### 2.5. Thermal Conductivity

The thermal conductivity of a porous solid (λ) is the sum of the contributions coming from the solid phase ($\lambda_{s}$), gas phase ($\lambda_{g}$), radiation through the pores ($\lambda_{r}$), and heat convection in the gas phase ($\lambda_{c}$). $\lambda_{c}$ is negligible when the pore size is smaller than about 2–3 mm, which is considered the lower limit for conduction by convective currents of the gas trapped in cells. On the other hand, $\lambda_{r}$ is low for optically thick materials at room temperature. The conductivity of the gas phase can be decreased if the pore size is at the range of the average free path of air molecules ~70 nm due to the Knudsen effect. In the present case, dominant contributions will be $\lambda_{s}$ and $\lambda_{g}$.

λ recorded from PVA and PVA modified aerogels are displayed in Table 3. Within the experimental errors, the conductivities of both uncrosslinked and crosslinked samples increased in density following a power-law dependence. Relative low variations within the studied samples were found. It is noteworthy that measurements were carried out in a dried state to avoid the influence of water. It is well known that λ increases with moisture content. The aerogel moisture absorption can be minimized by applying a hydrophobic coating to the aerogel surfaces [34,35].

In the uncrosslinked aerogels, pure PVA (P5 sample) showed the lowest values (λ= 0.030 W/m·K) and the lowest apparent density ($\rho_{app}$ = 0.075 ± 0.005 g/cm^3^), whereas P5T3A3 presented the highest conductivity (λ= 0.046 W/m·K) as well as the highest density ($\rho_{app}$ = 0.144 ± 0.009 g/cm^3^). Despite the density dependence, the large difference between pure PVA and the aerogel blends is attributed to the hydrogen bond interactions between the components (PVA, TA, SA) that can create a continuous thermal network, increasing heat transfer [36]. Alginate is characterized by a thermal conductivity around 0.06 W/m·K, a consequence of the presence of carboxylic and hydroxyl groups on its skeleton. Increasing alginate content will also increase the aerogels’ conductivity (Table 3).

As expected, the thermal conductivity increased after crosslinking because the bonds increased the connections between the polymer chains, creating additional heat transfer pathways. Additionally, polymer chains became closer to each other, favoring the heat transfer. In particular, it was observed that thermal conductivity increased as the SA content increased in all cases, whereas TA did not affect it. The high chain interconnection achieved after crosslinking justifies the high thermal conductivity in the crosslinked aerogel blends [37].

### 2.6. Thermal Degradation

**Figure 5:**
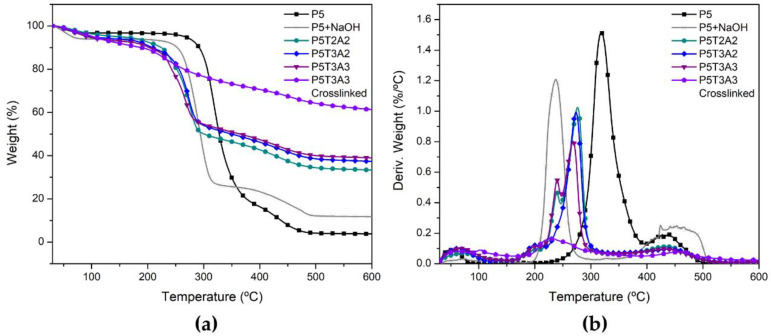
(**a**) TGA weight loss; (**b**) Derivative thermogravimetric curves of PVA/TA/SA aerogels. For the sake of clarity, only P5T3A3 crosslinked aerogel has been represented. See Figure S4 for the rest of the studied samples.

Figure 5 shows the thermogravimetric analysis results of PVA and PVA-modified aerogels. As NaOH was added to the precursor solutions to avoid earlier precipitation, an additional PVA-NaOH aerogel was prepared to determine the possible effect of NaOH on thermal degradation. The onset temperature ($T_{onset}$) was obtained according to the standard ISO 11358-1, which is defined as the intersection point between the starting-mass baseline and the tangent to the TGA curve at the maximum gradient point ($Td_{max}$). The temperature at maximum weight loss rate ($dW/dT_{max}$) and the residue at 600 °C (W_R_) are summarized in Table 4.

Due to many hydrophilic groups, the first drop in weight at 100 °C can be assigned to the moisture absorbed in the aerogel structure. The main weight loss in the PVA-based aerogels occurred between 170 to 310 °C, related to the loss of side groups and low molecular weight substances due to chain scission. A third step occurred between 355 and 430 °C, related to the breakdown of the polymer chain and cyclization, resulting in a decrease in dW/dT. As proposed by Cheng et al. [38], NaOH acts as a base catalyst, accelerating the thermal decomposition of pure PVA and increasing the amount of residue due to a change in the degradation mechanism through chain stripping. However, in our case, we did not observe a significant change in the degradation rate. The presence of SA and TA decreased the onset of decomposition because both materials began to degrade earlier than PVA.

Moreover, TA showed a clear impact on slowing down the thermal decomposition rate. The decomposition rate decreased from 1.5 to 0.8%/°C in the samples containing the largest amount of TA because it is an organic intumescent char-forming substance. Another effect was the increase in the residue after combustion. Irrespective of the amount of SA, the char increased in parallel to the increase in TA content (see Table 4).

In particular, sample P5T3A3 showed the lowest dW/dT_max_ and the highest amount of residue. It can be concluded that due to its conversion to graphitized forms, TA plays a major role in the formation of a charred residue barrier.

Crosslinking modified the degradation path leading to a more continuous degradation in which the main weight loss step, characteristic of PVA, almost disappeared. After crosslinking, the remarkable decrease in the weight loss rate clearly indicated a significant improvement in crosslinked aerogels’ thermal stability. In addition, the residual weight was almost doubled as compared to uncrosslinked samples, reaching 61% of the initial mass, implying that a stable and dense char layer had been created.

### 2.7. Combustion Behavior

**Figure 6:**
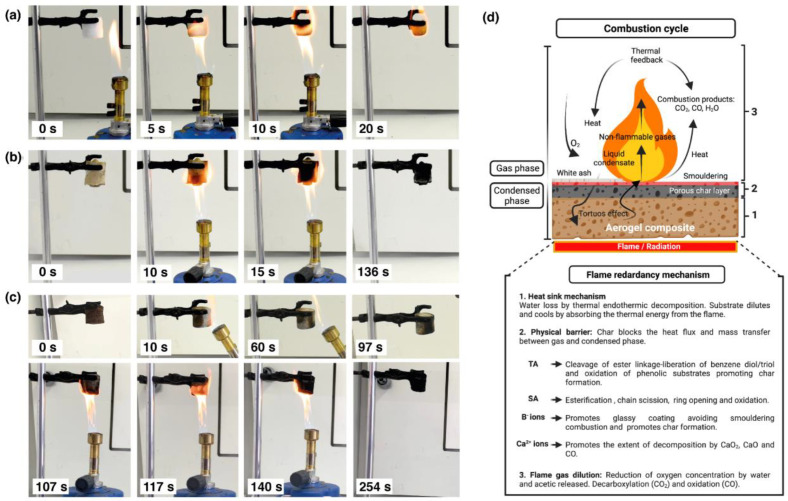
Flame behavior of (**a**) P5, (**b**) P5T3A3 uncrosslinked, and (**c**) crosslinked aerogels under Bunsen burner. (**d**) Diagrammatic scheme of combustion cycle and the flame retardant mechanism (Created with Biorender.com Agrmt. No.HF242VFEC0).

The flame behavior of aerogels was first qualitatively observed through vertical burning. Figure 6 shows the behavior of representative aerogel samples under a Bunsen torch. The PVA aerogel immediately ignited with a remaining flame until total sample consumption. (Figure 6a) A significant reduction of the initial volume, as well as no char residue, was observed.

In contrast, PVA/TA/SA aerogels exhibited a self-extinguishing performance, showing lower changes in volume and self-supporting char (see Videos S1–S4). The crosslinked aerogels (Figure 6c) showed a higher fire resistance in comparison to uncrosslinked ones (Figure 6b). The latter required about 2 min for complete combustion while the former lasted more than 4 min. This test demonstrated the flame retardant effect of TA/SA addition in the PVA aerogel structure and its improvement after crosslinking. Figure 6d illustrates the proposed flame retardant mechanism of these aerogels.

The combustion behavior of P5/TA/SA aerogels was analyzed using a cone calorimeter. Table 5 lists the results obtained for the most relevant parameters of this test, namely the time to ignition (TTI), heat release rate (HRR), peak of heat release rate (PHRR), time to peak of heat release rate (TTPHRR), total heat release (THR), and fire growth rate (FIGRA). The PHRR was taken from the first peak of the HRR curves. This set of parameters made it possible to evaluate the behavior of the aerogels under the external applied heat flux of 50 kW/m^2^, which corresponds to a fully developed fire scenario.

**Figure 7:**
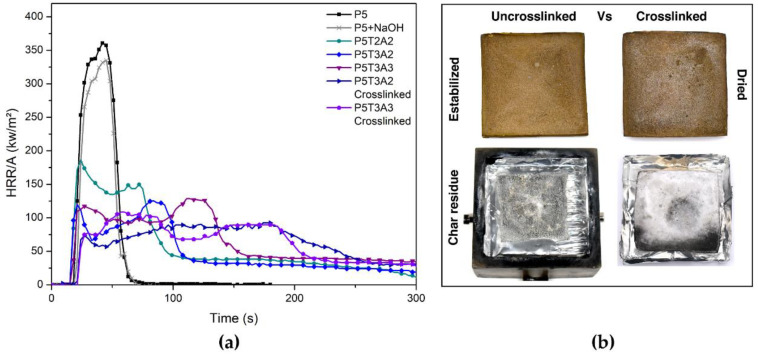
(**a**) Main representative HRR curves from uncrosslinked and crosslinked PVA/TA/SA aerogels; (**b**) digital photographs from P5T3A3 uncrosslinked and crosslinked aerogels before and after combustion at 50 kW/m^2^.

The HRR measures the flame spread rate, whereas the fire’s intensity is related to the peak of HRR. The representative HRR curves of the different aerogels tested are shown in Figure 7 (all HRR curves are shown in Figure S5). PVA is a highly combustible polymer; therefore, its aerogels are characterized by a spontaneous ignition followed by a rapid and intense burning, resulting in a very high value of PHRR (361 kW/m^2^). This value is similar to those reported by other researchers [39,40,41,42]. This high HRR is a consequence of the large porosity that increases the effective surface exposure to radiation and facilitates the thermal radiation through the internal core and bottom sample surface.

The presence of NaOH caused a minimal decrease in the PHRR value (Figure 7). When exposed to the external heat flux of 50 kW/m^2^, P5-NaOH aerogel immediately ignited, developing a vigorous flame without significant differences from those observed for P5. This result contrasts with the behavior reported by Cheng et al. [36], who found a notable decrease for similar contents of NaOH (0.5 g). It is speculated that the difference could be attributed to the low molecular weight of the PVA employed (Mw 13,000–23,000 vs. MW = 72,000 g/mol), which also yielded almost a double value in the PHRR value in comparison to the one tested here. However, in line with their observations, the residue left by the NaOH-modified aerogel was much higher than that of pure PVA, indicating the alkaline catalysis of char, favoring the generation of double bonds within the polymer backbone and the cyclization to yield aromatic char structures.

In comparison, the modified aerogels showed a notable and continuous decrease in the PHRR values. The presence of TA led to a 40% reduction in the sample P5T2A1 (TA/PVA ratio of 0.4) and a 55% reduction for the P5T3A1 aerogel (TA/PVA ratio of 0.6). As shown in Table 5, the presence of SA also contributed to a further decrease in PHRR, as the amount of SA increased in the aerogel structure, reaching a three-fold reduction in the case of P5T3A3. Compared to TA, the flame retardant efficiency of SA is considered to be lower. The shape of the HRR curves exhibited a different trend changing from a single peak for the P5 aerogel to a double peak signal for the blends. The second peak appearing for a longer time is associated with a charring process induced by TA under pyrolytic conditions with a contribution of the SA surface oxidation, which slowly degraded without the presence of flame (smoldering). The formation of a protective layer caused the decrease in the HRR from the first peak, whereas the rupture of the protective layer was the origin of the second peak located for a longer time. The effective total heat release (THReff) and the fire growth (FIGRA) indicate that the modified aerogels had lower flammability than the reference. The THReff reflects the total heat released by the samples once normalized by the different mass of each specimen. It decreased when TA and SA were incorporated showing, in general, slight differences between the samples. The samples containing a higher relative PVA/TA ratio (P5T3 compositions) showed an average FIGRA of 4.96 kW/s·m^2^. In contrast, those containing a lower PVA/TA ratio (P5T2 compositions) showed, on average, a FIGRA of 8.2 kW/s·m^2^. This fact corroborates the protective effect of TA against fire.

In regard to the formed residue, the data obtained shows a clear increase from an almost negligible amount for the PVA case to values higher than 10%w in the modified aerogels. Moreover, the presence of a growing amount of SA within the aerogels generated a parallel increase in the residue. Decomposition of SA generated CO_2_ and CO as well as porous char made of short-chain oligomers and monomers that could undergo crosslinking by oxidation, strengthening the char [10].

Furthermore, as was observed in the thermal degradation section, B(OH)_4_^−^ and Ca^2+^ crosslinking increased the fire resistance of all aerogels by decreasing PHRR and FIGRA and increasing the amount of char. In our opinion, the white residue is most likely the result of boric acid oxidation after a long exposure time at high temperatures [43]. Likewise, it should be considered that crosslinked samples were tested completely dry to avoid the effect of the different moisture absorptions in the fire parameters. Evidently, high moisture content will increase the ignition time and decrease the PHRR and FIGRA to a higher level than the values presented in Table 5. The composition with higher TA content obtained a 40% reduction in the PHRR and around 50% in FIGRA values. The residual weight exhibited a three-fold increase in regard to the uncrosslinked ones. On this basis, the aerogels with higher SA and TA amounts are considered the most promising composition as flame retardant systems.

## 3. Conclusions

In the present research, new aerogel blends with enhanced thermo-mechanical and resistance to fire have been successfully developed through a simple method, using alginate and tannic acid as natural modifiers for PVA. The hydrogen attraction between the components, especially between TA and PVA, is thought to be the main reason for the increased properties. Ca^2+^ and B(OH)_4_^−^ ions permeated the aerogel structure, creating a crosslinked network and making the aerogel stronger. However, the internal ice-template microstructure was not substantially changed as deduced by SEM images. When compared to the pure PVA aerogel, the specific Young’s modulus and yield strength increased nine-fold in the modified aerogels and up to six-fold after the crosslinking and drying process. TGA testing showed a decrease in the onset of degradation and much lower degradation kinetics mainly because of an enhanced barrier created by the TA.

Fire properties were also significantly enhanced as compared to pure PVA aerogel, with a three-fold and five-fold decrease in the PHRR in the uncrosslinked and crosslinked samples, respectively. This work shows a way to obtain low-density foam-like materials with enhanced mechanical properties and high fire resistance by using natural substances as modifiers.

## 4. Materials and Methods

### 4.1. Materials

Poly (vinyl alcohol) (Mw 72,000 g/mol, 85–89% hydrolyzed) was purchased from Panreac Química S.L.U. (Castellar del Vallès, Barcelona, Spain). Sodium alginate was received from Boter S.L. (Badalona, Barcelona, Spain). Tannic acid (Mw 1701.2 g/mol) and Boric acid EMSURE ACS (Mw 61.83 g/mol, 1.14 g/cm^3^) was supplied by Merck (Mollet del Vallés, Barcelona, Spain). Sodium hydroxide in the form of pellets was obtained from Riser S.L. (Les Franqueses del Vallès, Barcelona, Spain). Calcium chloride (anhydrous powder, ≥97%, Honeywell Fluka, Mw 110.978, 2.150 g/cm^3^) was purchased from Thermo Fisher Scientific Inc. (Cornellà de Llobregat, Barcelona, Spain). All reagents were used as received without further purification.

### 4.2. Aerogel Preparation

To prepare gel precursors, 5 g of PVA were first dispersed in 50 mL DI water at 90 °C under continuous magnetic stirring for about 2 h. The required amount of SA was then hydrated in 35 mL DI water using a Heidolph overhead stirrer (Heidolph Instruments GmbH, Schwabach, Germany) operating at 200 rpm. The respective grams of TA were dissolved in 15 mL of DI water by magnetic agitation (Agimatic-E, JP Selecta, Abrera, Barcelona, Spain). Once homogenized, NaOH (between 0.29 g to 0.88 g) was slowly added to the TA solution to reach a pH of 8.5, measured by pH analyzer PC 80+ (XS Instruments, Carpi, Modena, Italy). PVA and alginate gel precursors were mixed under mechanical stirring to obtain a homogeneous phase was obtained. Subsequently, the previously prepared TA-NaOH solution was slowly added to the PVA/SA blend. The resultant solution was transferred into cylindrical vials of 30 mm diameter for later use in measurements of density, porosity, water absorption, mechanical evaluation, microstructural evaluation, and thermal conductivity. Solutions were also poured into square molds (100 × 100 mm) for fire behavior analysis. The molds were immediately frozen in a solid CO_2_/ethanol bath. Finally, the frozen samples were freeze-dried for 4 days at −80 °C and 0.02 mbar in a Testlar Lyoquest lyophilizer (Terrassa, Barcelona, Spain).

#### 4.2.1. Post-Ion-Crosslinking Process

A solution of 12 g of CaCl_2_/12 g of B(OH)_3_ in 100 mL at 60 °C ethanol was prepared. The PVA/TA/SA aerogels were completely submerged in this solution for 90 min at RT. Samples were exhaustively washed with ethanol and then dried for 24 h in a −40 °C dew point Piovan T30 dehumidifier (Piovan S.p.A, Santa Maria di Sala, Venice, Italy).

#### 4.2.2. Characterization

The $\rho_{app}$ of samples was calculated following ISO 845 standard procedure. The mass was recorded from a semi-micro analytical balance (0.1 mg/0.01 mg) (AUW120D Shimadzu, Cobos Precisión S.L., L’Hospitalet de Llobregat, Barcelona, Spain), and the sample volume was measured by a digital vernier caliper (Mitutoyo, CD-20DC, Andover, Hampshire, UK). Before measurement, a minimum of five samples were dried overnight at 60 °C under 70 mbar in a vacuum drying oven (Vaciotem-TV, JP SelectaTM, Abrera, Barcelona, Spain). Then the samples were stabilized under a controlled atmosphere (50% relative humidity), which was created by placing a solution of 37 mL of sulfuric acid in 100 mL of DI water in hermetic containers inside an air oven (Heraeus Deutschland GmbH & Co, Hanau, Hesse, Germany) at 25 °C.

The porosity, defined as the volume fraction of voids, was calculated according to the equation:

(1) $$P\left(\%\right)=\left(1-\frac{\rho_{app}}{\rho_{x}}\right)\times 100$$

where the theoretical porosity $P_{th}$ is obtained when $\rho_{x}$ = theoretical density ($\rho_{th}$), expressed as follows:

(2) ρth=1∑i=1n(wiρi)

where $w_{i}$ is the mass fraction of each component (PVA, TA, SA, and NaOH), and $\rho_{i}$ refers to the respective densities.

The $P_{exp}$ is obtained when $\rho_{x}$ = experimental density ($\rho_{exp}$), calculated by the following formula:

(3) $$\rho_{exp}=\frac{W_{c+s}-W_{c}}{V_{s}}$$

where $W_{c+s}$ corresponds to the weight of the chamber with the sample, $W_{c}$ corresponds to the empty chamber weight, and $V_{s}$ corresponds to the volume of solid skeleton obtained through the helium pycnometer (Micromeritics, AccuPyc 1330, L’Hospitalet de Llobregat, Barcelona, Spain).

The microstructure analyses were conducted using a field emission scanning electron microscope (Jeol, JSM-7001F, Akishima, Tokyo, Japan). Before being analyzed, the aerogels were cryofractured using liquid nitrogen and sputter-coated with 80% platinum and 20% palladium to make them conductive.

For the Fourier transform infrared analysis (FTIR), a Nicolet 6700 spectrophotometer (Thermo Fisher Scientific, Waltham, MA, USA) was set in the attenuated total reflectance (ATR) mode. The spectral results were based on 30 sample scans with a 1 cm^−1^ resolution ranging between 4000 and 400 cm^−1^.

The compressive behavior was evaluated following ISO 604 standard procedure. The values were recorded by a universal testing machine (Galdabini, Cardano al Campo, Varese, Italy) using a load cell of 5 kN and a crosshead rate of 1 mm/min. The Young’s modulus was obtained from the slopes of the initial region of the stress–strain curves. The yield stress was taken as the stress at the intersection between the tangent lines corresponding to the elastic region and the tangent line of the stress plateau segment. The corresponding specific values were obtained by dividing Young’s modulus and stress by the samples’ density. Due to the influence of moisture content on the mechanical properties, uncrosslinked and crosslinked samples were conditioned and stabilized under previously described conditions. After the compression test, their moisture content was measured with a Mettler Toledo moisture analyzer HE53 (Greifensee, Zürich, Switzerland) by heating the samples for 30 min at 160 °C.

The λ of aerogels was determined experimentally using a Hot Disk model analyzer based on the Modified Transient Plane Source (TPS) (C-Therm, TCi Thermal Conductivity Analyzer, Fredericton, NB, Canada). Experiments were performed with a sensor radius of 3.189 mm. The power level was 60 mW, and test and cooling times were 2.7 and 90 s, respectively. The cylindrical samples’ top and bottom surfaces were flattened using a polishing machine (Struers DAP-7, Ballerup, Copenhagen, Denmark), and the final sample dimensions were D = 30 mm, h = 25 mm.

The thermal stability of aerogels was conducted on a TGA/DSC 1 instrument (Mettler Toledo, Columbus, OH, USA). Approximately 10 mg portions of the samples were placed on aluminum pans and then heated at 10 °C/min from 30 °C to 600 °C under a nitrogen atmosphere. Three replicas per composition were tested.

The fire behavior of aerogels was tested using a cone calorimeter (Ineltec, BECC, Tona, Barcelona, Spain) following ISO 5660. Square monolith samples with an area of 100 mm × 100 mm and a thickness of approximately 10 mm were placed inside a steel support and exposed to an external heat flux of 50 kW/m^2^.

## Figures and Tables

**Table 1:** Apparent densities and experimental and theoretical porosity of PVA/TA/SA uncrosslinked and crosslinked aerogels.

Sample	Uncrosslinked			Crosslinked		
	$\rho_{app}\;(g/{\mathrm{cm}}^{3})$	$P_{exp}\;(\%)$	$P_{th}\;(\%)$	$\rho_{app}\;(g/{\mathrm{cm}}^{3})$	$\rho_{app\left(Dried\right)}\;(g/{\mathrm{cm}}^{3})$	$P_{exp}\;(\%)$
P5	0.075 ± 0.005	94.5	94.1	-	-	-
P5T2A1	0.157 ± 0.023	89.2	88.9	0.314 ± 0.048	0.244 ± 0.047	69.5
P5T2A2	0.163 ± 0.019	88.9	88.7	0.415 ± 0.020	0.300 ± 0.004	75.2
P5T2A3	0.169 ± 0.012	88.6	88.5	0.359 ± 0.002	0.294 ± 0.008	79.1
P5T3A1	0.158 ± 0.034	89.1	89.1	0.258 ± 0.012	0.217 ± 0.001	83.4
P5T3A2	0.164 ± 0.023	88.9	88.8	0.247 ± 0.031	0.211 ± 0.020	81.2
P5T3A3	0.167 ± 0.006	88.9	88.7	0.283 ± 0.005	0.233 ± 0.005	84.3

Unless indicated, densities were obtained after stabilization at 25 °C and 50% RH. Porosities were obtained in the dried state.

**Table 2:** Mechanical compressive specific properties of PVA/TA/SA aerogels, with and without crosslinking. Samples crosslinked were tested in both dry and stabilized states.

Sample	Uncrosslinked		Crosslinked		Crosslinked-Dried	
	E*_sp_* (MPa cm^3^/g)	σy*_sp_* (MPa cm^3^/g)	E*_sp_* (MPa cm^3^/g)	σy*_sp_* (MPa cm^3^/g)	E*_sp_* (MPa cm^3^/g)	σy*_sp_* (MPa cm^3^/g)
P5	6.3 ± 0.8	0.25 ± 0.01	-	-	-	-
P5T2A1	31.9 ± 5.4	1.77 ± 0.67	37.2 ± 1.4	2.02 ± 0.36	56.3 ± 3.5	2.17 ± 0.04
P5T2A2	35.6 ± 4.3	1.86 ± 0.55	38.7 ± 5.2	2.65 ± 0.60	60.7 ± 5.6	5.42 ± 0.78
P5T2A3	26.7 ± 6.9	2.17 ± 0.61	45.7 ± 5.3	2.42 ± 0.38	162.5 ± 9.5	5.87 ± 0.76
P5T3A1	31.6 ± 4.3	1.50 ± 0.31	38.8 ± 2.1	1.96 ± 0.26	80.1 ± 6.6	5.75 ± 0.88
P5T3A2	57.1 ± 1.5	2.07 ± 0.18	89.7 ± 1.8	2.61 ± 0.76	130.4 ± 1.6	5.85 ± 0.03
P5T3A3	53.6 ± 3.6	2.45 ± 0.10	61.6 ± 5.6	2.58 ± 0.01	127.0 ± 5.1	5.15 ± 0.67

**Table 3:** Thermal conductivities of uncrosslinked and crosslinked aerogels in a dried state.

Sample	Uncrosslinked	Crosslinked
	λ (W/m·K)	λ (W/m·K)
P5	0.030 ± 0.002	-
P5T2A1	0.043 ± 0.001	0.054 ± 0.003
P5T2A2	0.044 ± 0.001	0.059 ± 0.010
P5T2A3	0.046 ± 0.002	0.060 ± 0.009
P5T3A1	0.043 ± 0.001	0.049 ± 0.001
P5T3A2	0.044 ± 0.001	0.051 ± 0.004
P5T3A3	0.046 ± 0.002	0.057 ± 0.004

**Table 4:** Thermal degradation of aerogels.

Sample	Uncrosslinked				Crosslinked			
	$T_{onset}$ (°C)	$Td_{max}$ (°C)	$dW/dT_{max}$ (%/°C)	W_R_ (%)	$T_{onset}$ (°C)	$Td_{max}$ (°C)	$dW/dT_{max}$ (%/°C)	W_R_ (%)
P5	297.3	317.4	1.52	3.8	-	-	-	-
P5 + NaOH	218.6	237.5	1.21	27.1	-	-	-	-
P5T2A1	178.9	277.1	1.07	33.2	214.2	231.6	0.158	52.3
P5T2A2	177.1	273.8	1.05	34.1	217.3	235.7	0.160	50.5
P5T2A3	177.3	273.6	0.92	34.2	211.7	232.0	0.208	59.9
P5T3A1	187.1	271.2	0.90	39.3	225.6	274.4	0.146	56.1
P5T3A2	181.5	272.7	0.99	37.3	215.4	239.1	0.145	61.1
P5T3A3	186.4	269.0	0.83	38.9	206.2	229.5	0.164	61.3

**Table 5:** Cone calorimeter parameters for the different aerogels under study. Crosslinked samples were tested in a dry state. Uncrosslinked samples were tested after stabilization at 25 °C and 50% RH.

Sample	Uncrosslinked						Crosslinked					
	TTI (s)	PHRR (kW/m^2^)	TTPHRR (s)	THReff (MJ/m^2^·g)	FIGRA (kW/s·m^2^)	W_R_ (%)	TTI (s)	PHRR (kW/m^2^)	TTPHRR (s)	THReff (MJ/m^2^·g)	FIGRA (kW/s·m^2^)	W_R_ (%)
P5	0	328.6	30	2.53	10.96	1.3	0	-	-	-	-	-
P5 + NaOH	0	287.4	30	2.08	10.04	18.4	0	-	-	-	-	-
P5T2A1	0	210.2	24	2.39	8.76	10.8	16	141.3	48	2.25	2.94	39.1
P5T2A2	0	186.6	24	2.12	7.78	12.9	8	132.7	33	1.81	4.02	46.3
P5T2A3	1	192.8	24	1.99	8.03	14.4	5	110.1	55	1.70	2.00	38.1
P5T3A1	0	128.4	24	2.10	5.35	13.0	6	93.3	33	1.83	2.83	40.4
P5T3A2	0	119.6	21	2.09	5.70	14.5	5	68.1	27	1.51	2.52	39.3
P5T3A3	1	112.5	24	2.05	4.69	16.4	5	83.7	30	1.62	2.79	37.0

## Data Availability

The datasets generated and/or analyzed during the current study are not publicly available because they belong to on-going research but are available from the corresponding author upon reasonable request.

## Supplementary Materials

The following supporting information can be downloaded at: https://www.mdpi.com/article/10.3390/gels8070419/s1, Table S1: Aerogel compositions; Figure S1: Aerogel monoliths for (a) fire test and (b) the rest of the characterizations; Figure S2: TA effect on the microstructure of (a) P5T2A3 vs. (b) P5T3A3 aerogels; Figure S3: FTIR comparison of all aerogels; Figure S4: Thermal degradation behavior of (a) uncrosslinked and (b) crosslinked aerogels; Figure S5: HRR curves of (a) uncrosslinked and (b) crosslinked PVA/TA/SA aerogels; The Videos S1–S4 are openly available at https://doi.org/10.5281/zenodo.6699900 (Video S1: P5 aerogel during vertical burning; Video S2: P5T3A3 uncrosslinked aerogel during vertical burning; Videos S3 and S4: Self-extinguishing performance of P5T3A3 crosslinked aerogel (parts 1 and 2, respectively).

## References

[1] Verma A., Thakur S., Goel G., Raj J., Gupta V.K., Roberts D., Thakur V.K. (2020). Bio-Based Sustainable Aerogels: New Sensation in CO_2_ Capture. Curr. Res. Green Sustain. Chem..

[2] Mao J., Iocozzia J., Huang J., Meng K., Lai Y., Lin Z. (2018). Graphene Aerogels for Efficient Energy Storage and Conversion. Energy Environ. Sci..

[3] Wu Y., Wang X., Liu L., Zhang Z., Shen J. (2021). Alumina-Doped Silica Aerogels for High-Temperature Thermal Insulation. Gels.

[4] Wang X., Xie P., Wan K., Miao Y., Liu Z., Li X., Wang C. (2021). Mechanically Strong, Low Thermal Conductivity and Improved Thermal Stability Polyvinyl Alcohol-Graphene-Nanocellulose Aerogel. Gels.

[5] Wang L., Sánchez-Soto M., Maspoch M.L. (2013). Polymer/Clay Aerogel Composites with Flame Retardant Agents: Mechanical, Thermal and Fire Behavior. Mater. Des..

[6] Wang H., Cao M., Zhao H.-B., Liu J.-X., Geng C.-Z., Wang Y.-Z. (2020). Double-Cross-Linked Aerogels towards Ultrahigh Mechanical Properties and Thermal Insulation at Extreme Environment. Chem. Eng. J..

[7] Zhou L., Zhai S., Chen Y., Xu Z. (2019). Anisotropic Cellulose Nanofibers/Polyvinyl Alcohol/Graphene Aerogels Fabricated by Directional Freeze-Drying as Effective Oil Adsorbents. Polymers.

[8] Li X.-L., Chen M.-J., Chen H.-B. (2019). Facile Fabrication of Mechanically-Strong and Flame Retardant Alginate/Clay Aerogels. Compos. Part B Eng..

[9] Chen H.-B., Wang Y.-Z., Sánchez-Soto M., Schiraldi D.A. (2012). Low Flammability, Foam-like Materials Based on Ammonium Alginate and Sodium Montmorillonite Clay. Polymer.

[10] Kabir I.I., Sorrell C.C., Mofarah S.S., Yang W., Yuen A.C.Y., Nazir M.T., Yeoh G.H. (2021). Alginate/Polymer-Based Materials for Fire Retardancy: Synthesis, Structure, Properties, and Applications. Polym. Rev..

[11] Nam S., Condon B.D., Xia Z., Nagarajan R., Hinchliffe D.J., Madison C.A. (2017). Intumescent Flame-Retardant Cotton Produced by Tannic Acid and Sodium Hydroxide. J. Anal. Appl. Pyrolysis.

[12] Chen Y.-N., Peng L., Liu T., Wang Y., Shi S., Wang H. (2016). Poly(Vinyl Alcohol)–Tannic Acid Hydrogels with Excellent Mechanical Properties and Shape Memory Behaviors. ACS Appl. Mater. Interfaces.

[13] Guan Y., Shao L., Dong D., Wang F., Zhang Y., Wang Y. (2016). Bio-Inspired Natural Polyphenol Cross-Linking Poly(Vinyl Alcohol) Films with Strong Integrated Strength and Toughness. RSC Adv..

[14] Cheng Z., DeGracia K., Schiraldi D.A. (2018). Sustainable, Low Flammability, Mechanically-Strong Poly(Vinyl Alcohol) Aerogels. Polymers.

[15] Grant G.T., Morris E.R., Rees D.A., Smith P.J.C., Thom D. (1973). Biological Interactions between Polysaccharides and Divalent Cations: The Egg-Box Model. FEBS Lett..

[16] Ochiai H., Shimizu S., Tadokoro Y., Murakami I. (1981). Complex Formation between Poly(Vinyl Alcohol) and Borate Ion. Polymer.

[17] Erel-Unal I., Sukhishvili S.A. (2008). Hydrogen-Bonded Multilayers of a Neutral Polymer and a Polyphenol. Macromolecules.

[18] Ni X., Ke F., Xiao M., Wu K., Kuang Y., Corke H., Jiang F. (2016). The Control of Ice Crystal Growth and Effect on Porous Structure of Konjac Glucomannan-Based Aerogels. Int. J. Biol. Macromol..

[19] Sun M., Sun H., Wang Y., Sánchez-Soto M., Schiraldi D.A. (2018). The Relation between the Rheological Properties of Gels and the Mechanical Properties of Their Corresponding Aerogels. Gels.

[20] Joseph J., Jemmis E.D. (2007). Red-, Blue-, or No-Shift in Hydrogen Bonds: A Unified Explanation. J. Am. Chem. Soc..

[21] Kumar A., Lee Y., Kim D., Rao K., Kim J., Park S., Haider A., Lee D., Han S. (2017). Effect of Crosslinking Functionality on Microstructure, Mechanical Properties, and in Vitro Cytocompatibility of Cellulose Nanocrystals Reinforced Poly(Vinyl Alcohol)/Sodium Alginate Hybrid Scaffolds. Int. J. Biol. Macromol..

[22] Li N., Yang X., Liu W., Xi G., Wang M., Liang B., Ma Z., Feng Y., Chen H., Shi C. (2018). Tannic Acid Cross-Linked Polysaccharide-Based Multifunctional Hemostatic Microparticles for the Regulation of Rapid Wound Healing. Macromol. Biosci..

[23] Kim S., Kwak S., Lee S., Cho W.K., Lee J.K., Kang S.M. (2015). One-Step Functionalization of Zwitterionic Poly[(3-(Methacryloylamino)Propyl)Dimethyl(3-Sulfopropyl)Ammonium Hydroxide] Surfaces by Metal–Polyphenol Coating. Chem. Commun..

[24] Helmiyati, Aprilliza M. (2017). Characterization and Properties of Sodium Alginate from Brown Algae Used as an Ecofriendly Superabsorbent. IOP Conf. Ser. Mater. Sci. Eng..

[25] Harahap M.R., Mauliza N., Asmara A.P., Lestari E.C., Afriani W. (2020). The Effect of Seaweed Combination on the Extract of Robusta Coffee (Coffea Robusta) Waste Extract in Producing Facial Mask Products. Biomedika.

[26] Hu T., Liu Q., Gao T., Dong K., Wei G., Yao J. (2018). Facile Preparation of Tannic Acid–Poly(Vinyl Alcohol)/Sodium Alginate Hydrogel Beads for Methylene Blue Removal from Simulated Solution. ACS Omega.

[27] Liu R., Ge H., Wang X., Luo J., Li Z., Liu X. (2016). Three-Dimensional Ag–Tannic Acid–Graphene as an Antibacterial Material. New J. Chem..

[28] Wu N., Niu F., Lang W., Xia M. (2019). Highly Efficient Flame-Retardant and Low-Smoke-Toxicity Poly(Vinyl Alcohol)/Alginate/Montmorillonite Composite Aerogels by Two-Step Crosslinking Strategy. Carbohydr. Polym..

[29] Abulateefeh S.R., Taha M.O. (2015). Enhanced Drug Encapsulation and Extended Release Profiles of Calcium–Alginate Nanoparticles by Using Tannic Acid as a Bridging Cross-Linking Agent. J. Microencapsul..

[30] Schmidt M.P., Siciliano S.D., Peak D. (2021). The Role of Monodentate Tetrahedral Borate Complexes in Boric Acid Binding to a Soil Organic Matter Analogue. Chemosphere.

[31] Sun L., Wang J., Liang J., Li G. (2020). Boric Acid Cross-Linked 3D Polyvinyl Alcohol Gel Beads by NaOH-Titration Method as a Suitable Biomass Immobilization Matrix. J. Polym. Environ..

[32] Abureesh M.A., Oladipo A.A., Gazi M. (2016). Facile Synthesis of Glucose-Sensitive Chitosan–Poly(Vinyl Alcohol) Hydrogel: Drug Release Optimization and Swelling Properties. Int. J. Biol. Macromol..

[33] Konidari M.V., Papadokostaki K.G., Sanopoulou M. (2011). Moisture-Induced Effects on the Tensile Mechanical Properties and Glass-Transition Temperature of Poly(Vinyl Alcohol) Films. J. Appl. Polym. Sci..

[34] Zhang Q., Wang X., Tao X., Li Z., Li X., Zhang Z. (2019). Polyvinyl Alcohol Composite Aerogel with Remarkable Flame Retardancy, Chemical Durability and Self-Cleaning Property. Compos. Commun..

[35] Toledo P.V.O., Petri D.F.S. (2019). Hydrophilic, Hydrophobic, Janus and Multilayer Xanthan Based Cryogels. Int. J. Biol. Macromol..

[36] Zhang L., Ruesch M., Zhang X., Bai Z., Liu L. (2015). Tuning Thermal Conductivity of Crystalline Polymer Nanofibers by Interchain Hydrogen Bonding. RSC Adv..

[37] Mu L., He J., Li Y., Ji T., Mehra N., Shi Y., Zhu J. (2017). Molecular Origin of Efficient Phonon Transfer in Modulated Polymer Blends: Effect of Hydrogen Bonding on Polymer Coil Size and Assembled Microstructure. J. Phys. Chem. C.

[38] Cheng Z.-H., Guo M.-L., Chen X.-Y., Wang T., Wang Y.-Z., Schiraldi D.A. (2021). Reduction of PVA Aerogel Flammability by Incorporation of an Alkaline Catalyst. Gels.

[39] Kang A.-H., Shang K., Ye D.-D., Wang Y.-T., Wang H., Zhu Z.-M., Liao W., Xu S.-M., Wang Y.-Z., Schiraldi D.A. (2017). Rejuvenated Fly Ash in Poly(Vinyl Alcohol)-Based Composite Aerogels with High Fire Safety and Smoke Suppression. Chem. Eng. J..

[40] Chen J., Jiang S.-D., Huang Z.-Q., Tang G., Hu Y. (2017). Self-Assembly of Hydroxyapatite with Polyelectrolyte as a Green Flame Retardant for Poly(Vinyl Alcohol). J. Fire Sci..

[41] Chen H.-B., Wang Y.-Z., Schiraldi D.A. (2014). Preparation and Flammability of Poly(Vinyl Alcohol) Composite Aerogels. ACS Appl. Mater. Interfaces.

[42] Shang K., Yang J.-C., Cao Z.-J., Liao W., Wang Y.-Z., Schiraldi D.A. (2017). Novel Polymer Aerogel toward High Dimensional Stability, Mechanical Property, and Fire Safety. ACS Appl. Mater. Interfaces.

[43] Rasmussen R.L., Morse J.G., Morse K.W., Meyers R.A. (2003). Main Group Elements. Encyclopedia of Physical Science and Technology.

