# Supplementary material for: Ice-Template Crosslinked PVA Aerogels Modified with Tannic Acid and Sodium Alginate
Source: Gels. 2022 Jul 5;8(7):419. doi: 10.3390/gels8070419 (PMC9321210; doi:10.3390/gels8070419)
Supplement: Supplementary file 1 [file gels-08-00419-s001.zip › gels-1785869-supplementary.pdf]

Supplementary material

Table S1. Aerogel compositions.

Sample	PVA		TA		SA		NaOH	
	g	(% total)	g	(% total)	g	(% total)	g*	(% total)
P5	5	100	0	0	0	0	0	0
P5T2A1	5	58	2	23	1	12	0.587	7
P5T2A2	5	52	2	21	2	21	0.587	6
P5T2A3	5	47	2	19	3	28	0.587	6
P5T3A1	5	51	3	30	1	10	0.881	9
P5T3A2	5	46	3	28	2	18	0.881	8
P5T3A3	5	42	3	25	3	25	0.881	7

*Grams required for reaching pH 8.5

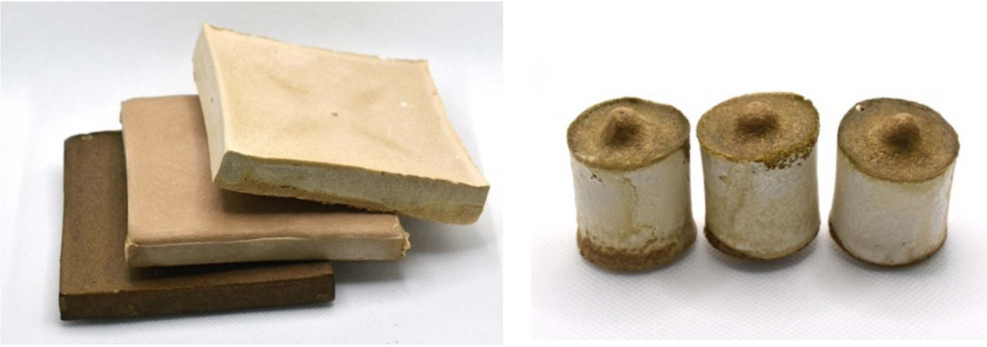

Figure S1. Aerogel monoliths for (a) Fire testing and (b) the rest of the characterizations.

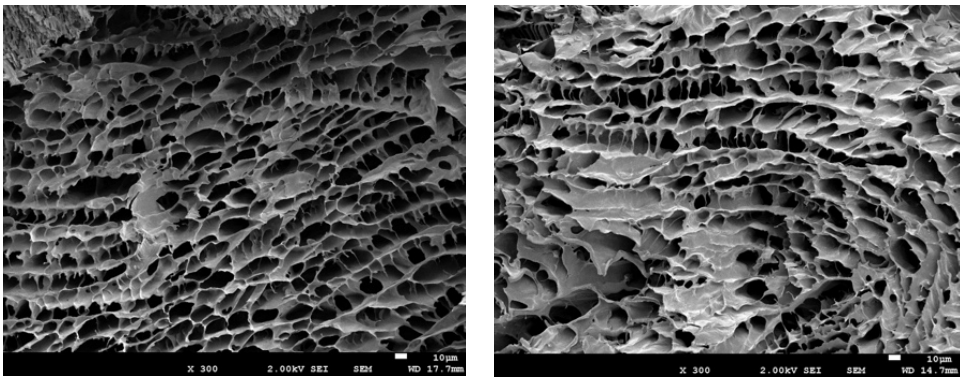

(a) (b)

Figure S2. TA effect on the microstructure of (a) P5T2A3 Vs. (b) P5T3A3 aerogels.

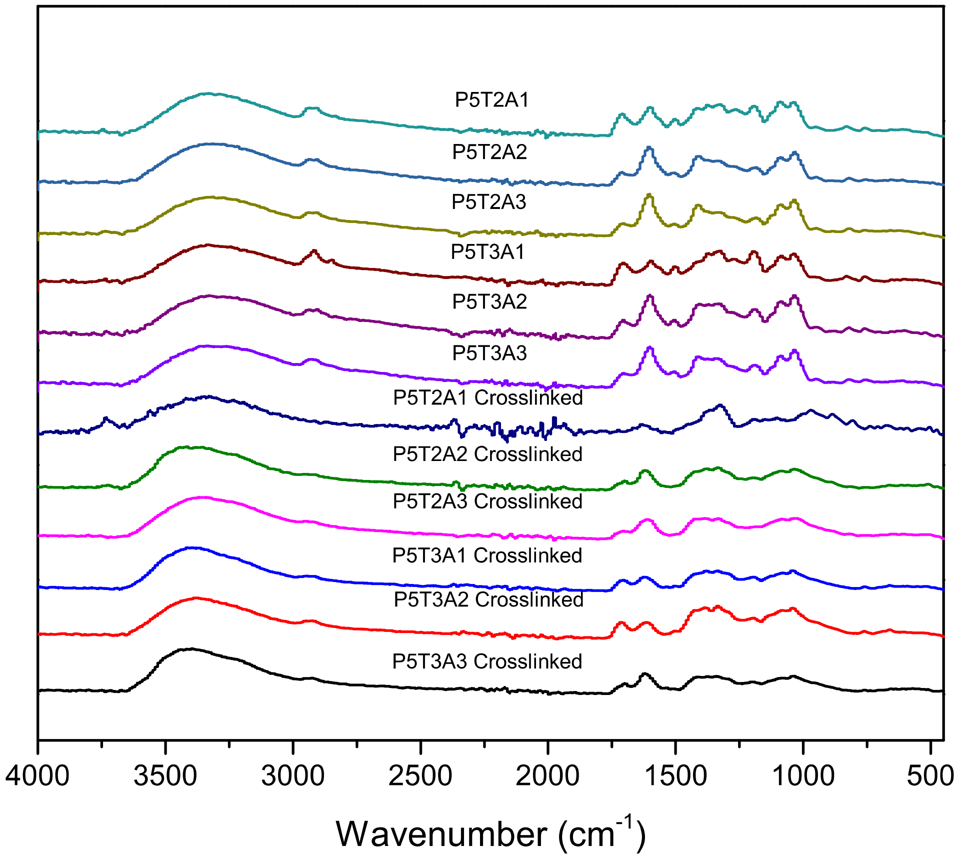

Figure S3. FTIR comparison of all the aerogels compositions.

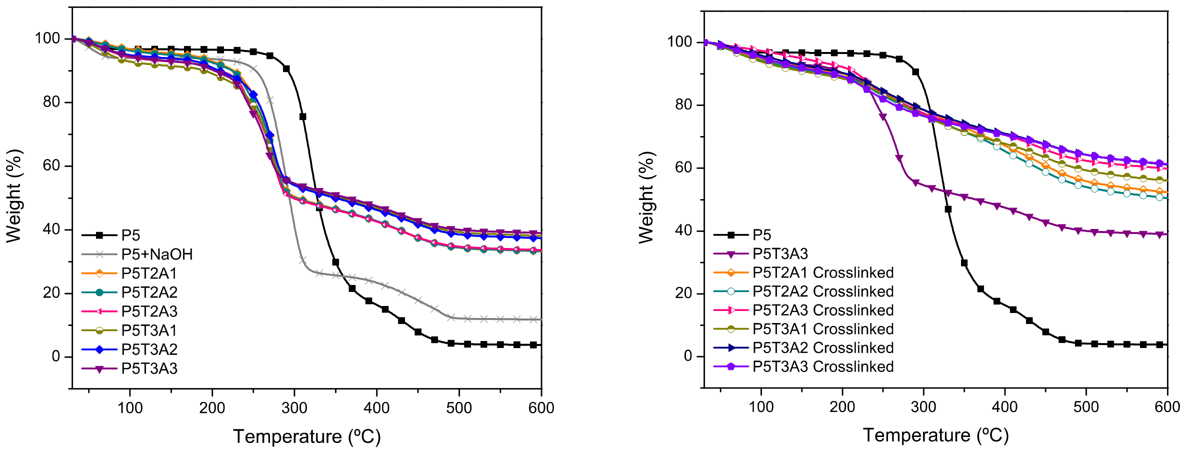

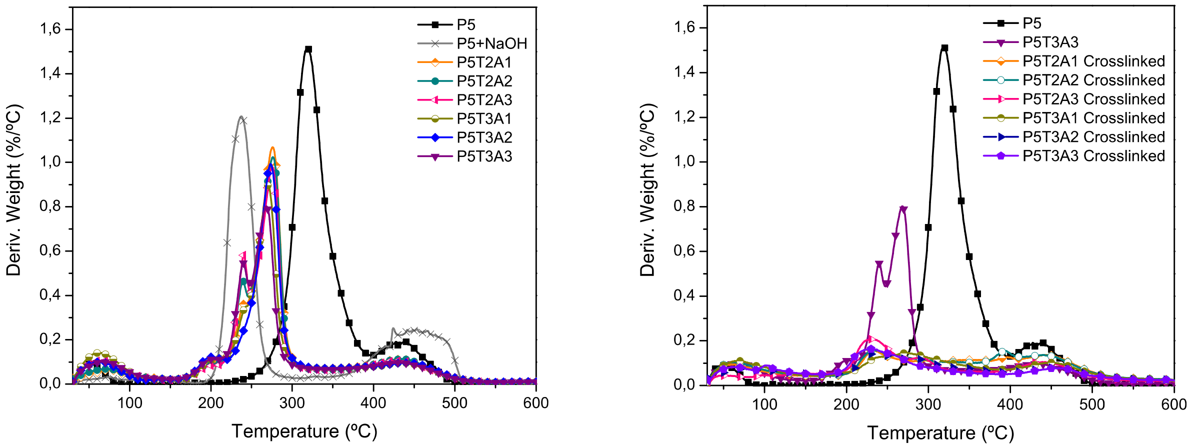

(a) (b)

Figure S4. Thermal degradation behavior of (a) uncrosslinked and (b) crosslinked aerogels.

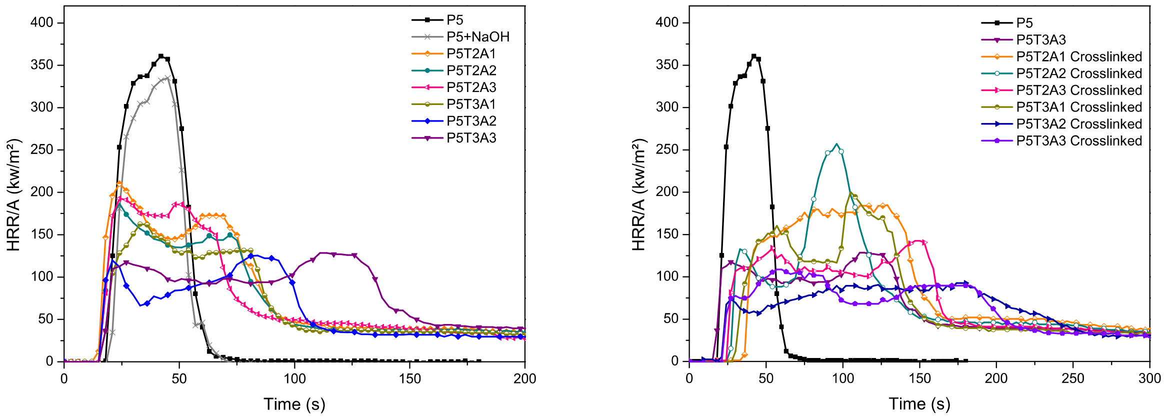

(a) (b)

Figure S5. HRR curves of (a) uncrosslinked and (b) crosslinked PVA/TA/SA aerogels.

Videos S1 to S4 can be found in the following link: https://doi.org/10.5281/zenodo.6699900

Video S1: P5 aerogel during vertical burning; Video S2: P5T3A3 uncrosslinked aerogel during vertical burning; Video S3 and S4: Self-extinguished performance of P5T3A3 crosslinked aerogel (parts 1 and 2, respectively).
